# Leveraging Machine Learning for Personalized Wearable Biomedical Devices: A Review

**DOI:** 10.3390/jpm14020203

**Published:** 2024-02-13

**Authors:** Ali Olyanasab, Mohsen Annabestani

**Affiliations:** 1 Institute for Integrated Circuits, Johannes Kepler University Linz, 4040 Linz, Austria; olyanasab.a@gmail.com; 2 Weill Cornell Medicine, Cornell University, New York, NY 10065, USA

**Keywords:** wearable devices, personalized, machine learning

## Abstract

This review investigates the convergence of artificial intelligence (AI) and personalized health monitoring through wearable devices, classifying them into three distinct categories: bio-electrical, bio-impedance and electro-chemical, and electro-mechanical. Wearable devices have emerged as promising tools for personalized health monitoring, utilizing machine learning to distill meaningful insights from the expansive datasets they capture. Within the bio-electrical category, these devices employ biosignal data, such as electrocardiograms (ECGs), electromyograms (EMGs), electroencephalograms (EEGs), etc., to monitor and assess health. The bio-impedance and electro-chemical category focuses on devices measuring physiological signals, including glucose levels and electrolytes, offering a holistic understanding of the wearer’s physiological state. Lastly, the electro-mechanical category encompasses devices designed to capture motion and physical activity data, providing valuable insights into an individual’s physical activity and behavior. This review critically evaluates the integration of machine learning algorithms within these wearable devices, illuminating their potential to revolutionize healthcare. Emphasizing early detection, timely intervention, and the provision of personalized lifestyle recommendations, the paper outlines how the amalgamation of advanced machine learning techniques with wearable devices can pave the way for more effective and individualized healthcare solutions. The exploration of this intersection promises a paradigm shift, heralding a new era in healthcare innovation and personalized well-being.

## 1. Introduction

Recent advances in the development of wearable devices have showcased the integration of machine learning algorithms to enable personalized health monitoring and intervention systems. These systems leverage advanced algorithms to process data from various sensors embedded in wearable devices, such as strain gauges, plastic optical fibers, actuators, and electrochemical sensors, to provide personalized health insights and interventions [1,2,3,4]. The use of machine learning allows these devices to classify and predict various health-related parameters, including blood glucose levels, blood pressure, stress levels, and physical activity, tailored to individual users’ needs and health conditions [5,6,7,8].

Moreover, the application of machine learning in wearable devices has extended to personalized healthcare monitoring for specific medical conditions, such as diabetes, sleep disorders, and neurological rehabilitation [9,10,11]. These systems utilize AI to analyze physiological signals, predict disease states, and recommend personalized interventions, contributing to improved disease management and patient outcomes. The integration of machine learning in these devices enables the real-time monitoring and interpretation of health-related data, leading to actionable insights and personalized recommendations for users’ health management.

**Figure 1:**
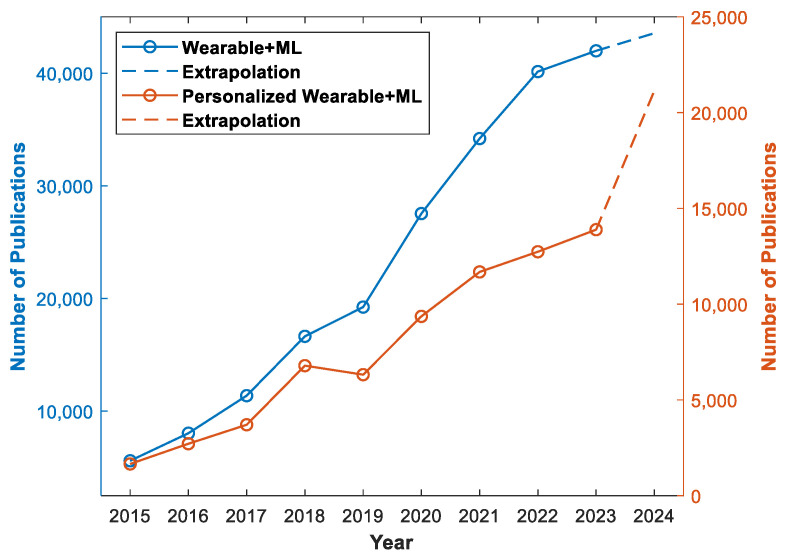
The blue plot illustrates number of publications on wearable devices utilizing machine learning and the orange plot is number of publications on personalized wearable devices using machine learning [15]. The keywords used were “wearable machine learning” and “personalized wearable machine learning”. The extrapolated data for 2024 were based on the number of publications up to 25 January 2024.

Furthermore, the development of personalized wearable devices has been driven by the need to provide tailored solutions for individuals with specific health conditions, such as urinary incontinence, panic attacks, and obsessive compulsive disorder [12,13,14]. Machine learning algorithms have been employed to detect and predict these conditions using sensor data from wearable devices, enabling early intervention and personalized support for individuals with these health challenges. These advancements highlight the potential of personalized wearable devices in addressing specific health needs and improving the quality of life for individuals with diverse health conditions. In recent years, there has been a notable surge in publications addressing the integration of machine learning methodologies in wearable devices. This upward trend, as evidenced by Figure 1, underscores the growing scholarly interest in exploring innovative applications and methodologies at the intersection of wearable technologies and machine learning algorithms. Simultaneously, a conspicuous thematic focus has emerged on personalized wearable devices, reflecting researchers’ increasing attention to the customization of wearable solutions. This indicates the massive potential of personalized wearable devices in patient care and underscores the ongoing evolution of contemporary academic research in wearable technology.

**Figure 2:**
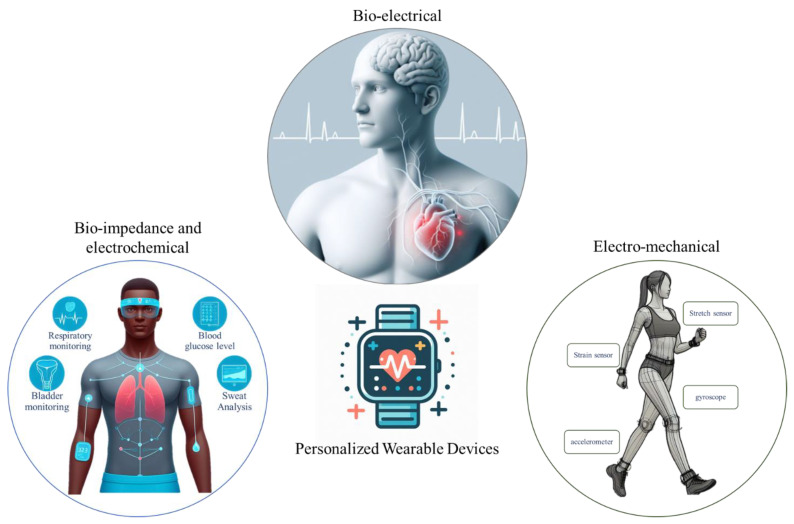
Illustration of three main categories of personalized wearable devices: bio-electrical, bio-impedance, and electro-chemical and electro-mechanical wearable devices. The figure was generated using the Bing AI chat bot.

The literature review was carried out by refining the papers through the SCOPUS, Nature, and IEEE-Xplore databases using the search terms “Personalized + Wearable + Machine Learning”. Eventually, the investigation methodically categorizes academic papers into three distinct thematic groups, as illustrated in Figure 2. The first category, designated as “Bio-electrical Wearable Devices”, delves into topics concerning electrical phenomena within biological systems, encompassing ECG, EEG, and EMG, among others. Papers in this category extensively explore the intricacies of bio-electrical processes and their implications. The second category, denoted as “Electro-Chemical and Bio-Impedance”, centers on the nuanced interplay between electrical and chemical processes in biological systems, with a specific emphasis on bio-impedance dynamics. Lastly, the third category, termed “Electro-Mechanical,” encompasses papers that investigate the intersection of electrical and mechanical phenomena within biological contexts. These papers explore electro-mechanical interactions, exemplified by technologies like gait sensors, stretchable sensors, and strain gauges. This tripartite categorization system provides a structured framework, enhancing the comprehension and navigation of the diverse themes presented in the journal papers.

## 2. Bio-Electrical Wearable Devices

Personalized wearable devices that utilize bio-electrical signals, such as ECG, EEG, and EMG, play a crucial role in revolutionizing healthcare and well-being. These devices offer the continuous and non-invasive monitoring of physiological signals, providing valuable insights into an individual’s health status and enabling personalized health management. The integration of machine learning algorithms with these wearable devices enhances their capabilities by enabling the analysis of complex bio-electrical data to detect anomalies, predict health conditions, and provide personalized recommendations. For instance, machine learning models can be trained to classify ECG signals for blood pressure estimation [6], EEG signals for emotion recognition [16], and EMG signals for gesture recognition [17]. This combination of personalized wearable devices and machine learning holds great promise in advancing preventive healthcare, early disease detection, and personalized treatment strategies, ultimately leading to improved patient outcomes and quality of life.

The integration of bio-electrical wearables into healthcare has ushered in a new era, with machine learning algorithms enhancing their capabilities across a myriad of applications. In the realm of personalized Parkinson’s disease management, LeMoyne et al. developed a groundbreaking system using the BioStamp nPoint. By adjusting the amplitude of the current applied to deep brain stimulation (4.0 mA, 2.5 mA, 1.0 mA, off), this multilayer neural network was capable of classifying tremor responses with 95% accuracy, demonstrating the potential for tailored interventions [10]. Moving to the domain of rehabilitation, LeMoyne et al. undertook a longitudinal investigation spanning 10 months. During this study, a smartphone affixed to the foot with an armband was employed to capture gyro data and transfer them to the cloud. The gathered data encompassed key metrics such as the maximum, minimum, mean, standard deviation, and coefficient of variation of the gyroscope signal. Subsequently, a support vector machine, facilitated by the Waikato Environment for Knowledge Analysis (WEKA), was employed to classify the gyroscope-acquired data in order to distinguish between the initial and final phases of the therapy regimen. The evaluation of the data demonstrates the effectiveness of the rehabilitation process [18].

Transitioning to cardiovascular health, Banerjee et al. proposed a methodology for blood pressure estimation using ECG data. Utilizing XGBoost for classification and an artificial neural network (ANN) for regression, their system achieved a mean error of 0.89 mm Hg. This application highlights the potential of lightweight ML algorithms in remote health monitoring, particularly in cardiovascular conditions [6]. An example of a wearable cardiovascular healthcare device is the system developed by Chiang et al. for predicting blood pressure (BP) and providing personalized lifestyle recommendations based on ECG data. Utilizing ECG, sleep, and physical activity data collected from smartwatches, the system uses ML models such as random forest and autoregressive integrated moving average (ARIMA) to predict BP and make lifestyle recommendations. The subjects experienced decreased BPs by 3.8 and 2.3 for systolic and diastolic BP. Furthermore, the system used Shapley values to identify lifestyle factors that contribute to high blood pressure [19]. Pramukantoro et al.’s real-time heartbeat monitoring system utilized the Polar H10 wearable device. For classification, they used SVM to categorize the data into five sections: normal, supraventricular, ventricular ectopic, fusion, and unknown. This exemplifies the potential for the accurate classification of heartbeats into five categories. The system’s use of RR interval data and Bluetooth low energy (BLE) enables real-time monitoring, showcasing bio-electrical wearables’ potential in cardiovascular health [20]. To show the unlimited features of an ECG signal, Maged et al. utilized ECG sensors in smartwatches to predict blood glucose levels in diabetic patients. Leveraging machine learning methods for regression, such as LGBM, GBR, AdaBoost, and linear and ridge regressors. and heart rate variability parameters, their system presented a novel approach to health monitoring. This application underscores the versatility of bio-electrical wearables in managing chronic conditions [21].

In the field of predictive healthcare and occupational safety, Shimazaki et al. employed supervised machine learning to prevent heat stroke in hot environments. Based on a personalized heat strain temperature (pHST) meter, their web survey-based automatic annotation system classified workers into thermal and non-thermal groups based on vital data, achieving an 85.2% accuracy in predicting heat stroke. This application highlights the potential of bio-electrical wearables in ensuring safety in challenging occupational environments [22].

Shifting focus to mental health, Campanella et al.’s stress detection system, utilizing physiological signals collected by the Empatica E4 bracelet and machine learning algorithms, introduces an application in stress management. The system collects physiological data through four sensors: a temperature sensor, accelerometer, photoplethysmogram (PPG) sensors, and electrodermal activity (EDA) sensors. Achieving an accuracy range of 70% to 79.17%, this study emphasizes the need for more extensive and diverse datasets to improve model accuracy, showcasing the potential of bio-electrical wearables in mental health [23]. Examining the real-time stress detection domain, Zhu et al. delved into EDA, ECG, and PPG signals from wearable devices. Utilizing six machine learning methods, including support vector machine (SVM) and k-nearest neighbors (KNNs), their stacking ensemble learning method achieved the best accuracy of 86.4% for EDA signals. This application demonstrates the potential of bio-electrical wearables in managing stress, offering real-time insights for users [7]. Tsai et al. devised a 7-day panic attack prediction model by leveraging data from a mobile app and a Garmin Vivosmart 4 smartwatch. The study encompassed 59 participants diagnosed with panic disorder (PD), and data on activity levels, heart rate, sleep patterns, anxiety, and depression scores were collected over a one-year period. Integrating questionnaires and additional physiological and environmental data, including the Air Quality Index (AQI), the researchers employed a random forest model, achieving prediction accuracies ranging from 67.4% to 81.3%. Crucial features such as Beck Anxiety Inventory (BAI), Beck Depression Inventory (BDI), State-Trait Anxiety Inventory (STAI), a Mini International Neuropsychiatric Interview (MINI), heart rate (HR), and deep sleep duration played pivotal roles in ensuring accurate predictions. This underscores the potential for early and personalized mental health interventions for patients diagnosed with panic disorder [13].

On the topic of gesture recognition, Ghaffar Nia et al. developed an artificial neural network (ANN) model with a few control parameters, achieving 98.9% and 93% accuracy in training and testing processes, respectively. The study focused on classifying EMG signals to control assistive devices for individuals with sensory-motor disorders. The ANN model’s application demonstrates the potential of machine learning algorithms in improving the accuracy and efficiency of EMG signal classification [17]. In another work, Avramoni et al. developed a sophisticated algorithm to detect pill intake using a smart wearable device with inertial measurement unit (IMU) sensors by evaluating the associated gestures. Employing supervised machine learning, the algorithm achieved over 99% accuracy in training and validation datasets and 100% accuracy in testing datasets. This application showcases the potential of bio-electrical wearables in gesture recognition and human–computer interaction [24].

Moving to neurological applications, Meisel et al. harnessed wristband sensor data and machine learning to develop a seizure forecasting system. The acquired signals includes EDA, blood volume pulse (BVP), temperature, and accelerometer data. The use of long short-term memory (LSTM) and 1D convolutional neural networks, optimized through grid searches, yielded promising results. The system not only showcased accurate predictions but also hinted at the potential for further improvement through individualized parameter tuning [25]. Transitioning to seizure detection, Jeppesen et al. developed a personalized seizure detection algorithm using patient-adaptive logistic regression machine learning (LRML). Utilizing a wearable ECG device and collecting heart rate variability (HRV) during a long-term video-EEG recording, the system achieved a 78.2% sensitivity and a 31% reduction in false alarm rates. This system contributes to the evolution of personalized healthcare interventions, showcasing the potential of bio-electrical wearables in neurological health [26].

Multiple studies have been conducted on sleep apnea detection and intervention. As an example, Ji et al. developed an airline point-of-care system for hybrid physiological signal monitoring, achieving high accuracy of 84–85% using a long short-term memory recurrent neural network (LSTM-RNN). The system detects electrocardiogram (ECG), breathing, and motion signals, with the diagnosis of sleep apnea-hypopnea syndrome (SAHS) as a key application. The hardware design includes ECG electrodes, flexible piezoelectric belts, and a control box, providing a low-cost, long-term monitoring solution for passengers during flights [27]. In another work, Mohan et al.’s exploration of deep learning and machine learning techniques for sleep apnea detection from single-lead ECG data emphasizes the potential of AI-based bio-signal processing. Their hybrid deep models achieved a sensitivity of 84.26%, a specificity of 92.27%, and an accuracy of 88.13%. This application showcases the potential for bio-electrical wearables in sleep monitoring and respiratory health [9].

The real-time emotion recognition system developed by Mai et al. using an ear-EEG-based on-chip device introduces a compact, battery-powered solution for emotion classification. Leveraging machine learning models such as SVM, MLP, and one-dimensional convolutional neural networks (1D-CNNs), this system utilizes Bluetooth low-energy wireless technology for data transmission, showcasing the potential for bio-electrical wearables in mental health applications [16].

**Figure 3:**
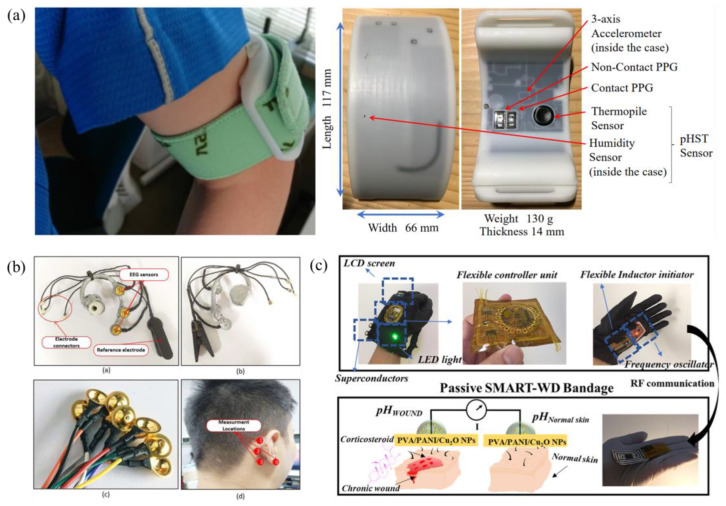
Schematics depicting the sensor setups for: (**a**) a system to prevent heat stroke in hot environments [22], (**b**) a real-time emotion recognition system [16], and (**c**) an intelligent wearable system for wound monitoring [28]. (**c**) is adapted by permission from [28]. Copyright 2022 American Chemical Society.

Figure 3 represents instances of the examined studies highlighting the utilization of bio-electrical wearable devices employing machine learning techniques.

Table 1 reviews recent research on personalized wearable bio-electrical devices from 2020 to 2023. The vast majority (86.6%) of the papers discuss devices customized to individual users. These personalized wearables leverage sensors like IMUs, ECGs, and EDAs to monitor physiological signals and detect conditions accurately, often with over 90% accuracy. For example, personalized devices using IMUs and ECGs can detect panic attacks, dehydration, wound healing, and Parkinson’s with over 90% accuracy [10,28,29,30]. Other applications include monitoring heart rate variability, sleep apnea, and stress levels [7,9,26]. The research shows a trend towards more personalized and accurate wearable sensors over time. While earlier papers from 2022 focus on non-personalized devices [6,23], the most recent 2023 studies emphasize personalized wearables [16,29]. In summary, Table 1 demonstrates personalized health monitoring wearables using sensors like IMUs and ECGs can provide highly accurate and customized detection for a variety of conditions.

## 3. Bio-Impedance and Electro-Chemical Wearables

Wearable devices utilizing electro-chemical and bio-impedance sensors have gained significant importance in healthcare and personalized health monitoring. These devices enable the non-invasive and continuous monitoring of various physiological parameters, such as glucose levels, electrolyte biomarkers, and tissue regeneration, providing valuable insights into an individual’s health status. The integration of machine learning algorithms with these wearable devices allows for the accurate interpretation of the collected data, leading to real-time health assessments and predictive analytics. The combination of wearable devices with electro-chemical and bio-impedance sensors, along with machine learning, holds great promise for revolutionizing personalized healthcare and improving overall well-being.

On impedance-based flow cytometry, Annabestani et al. introduced a sheath-free microfluidic system that employs machine learning to estimate the size and quantity of particles passing through the channel. This innovative approach utilizes a set of variables termed “Basis Impedances” and a memory-less version of a polynomial-based nonlinear auto-regressive with exogenous inputs (NARX) model to predict the total output impedance of multiparticle systems [31]. Shifting focus to respiratory monitoring, Rozo et al. developed machine learning models to assess thoracic bio-impedance (BioZ) measurements. Using SVM and CNN classifiers, transfer learning, and feature-based classification, they evaluated the impact of different breathing patterns on model performance [32].

Continuing the exploration of non-invasive health monitoring, Chahine et al. developed a wearable system utilizing electromagnetic sensors and artificial intelligence to non-invasively monitor glucose levels. This comprehensive system integrates environmental and physiological sensors to account for temperature, humidity, sweat, and motion effects, achieving high fidelity in tracking glucose variations with low error and good prediction accuracy [33]. Following the topic, Islam et al. designed a non-invasive glucose monitoring system using PPG and galvanic skin response (GSR) sensors, implementing a deep learning algorithm for improved prediction accuracy. The system collected data from 10 volunteers over 2 days and 15 patients over 1 day, using a total of 210 sample data points for training and testing the deep learning model. The deep learning model consisted of three stages: feature extraction, global average pooling, and regression. The results showed that the predicted blood glucose levels were accurate, with 80% of the training data and 40% of the testing data falling within acceptable error margins [5]. Another compelling area of research revolves around initiatives dedicated to wound monitoring and tissue regeneration. Kalasin et al. developed a contactless wearable system. This system, incorporating AI-enabled sensors and advanced wound dressing bandages, utilized an artificial neural network algorithm and a pH-responsive mechanism for wound monitoring. The integration of these elements showcases the potential for bio-electrical wearables in advanced healthcare applications [28].

Advancing the field of non-invasive sweat sensors, Sankhala et al. introduced a platform using electrochemical impedance spectroscopy and machine learning to report glucose concentrations. Employing ensemble and decision tree regression algorithms with k-fold cross-validation, the system optimizes models and prevents overfitting. The machine learning algorithm accurately interprets the progression trend of glucose levels, making it a valuable tool for lifestyle management [3]. As another example on sweat analysis, Wang et al. developed a patch with printed electrochemical sensors to monitor sweat biomarkers and predict core body temperature using machine learning algorithms. The system employs printed sensor patches with integrated microfluidics, utilizing machine learning to predict core body temperature based on real-time sweat biomarker measurements [34]. Nyein et al. developed a microfluidic patch for continuous sweat analysis during rest. The patch collects sweat from different body sites, enabling noninvasive monitoring of sweat rate, pH, and chloride levels. The study emphasizes machine learning for real-time data analysis. The patch’s potential for continuous metabolite monitoring makes it a promising tool for health and fitness applications [35]. Khosravi et al. developed a flexible electrochemical glucose sensor screen-printed onto a textile substrate, demonstrating a linear response in the range of 20–1000 µM of glucose concentration with high sensitivity (18.41 µA mM^−1^ cm^−2^, R2 = 0.996). The sensor showed high selectivity toward glucose and excellent stability over 30 days of storage. The study evaluated the successful immobilization of glucose oxidase and the sensor’s response to repeated glucose measurements [36].

The exploration of skin hydration levels by Liaqat et al. introduced a hybrid algorithm combining machine learning and deep learning methods. The collection of data from various postures and fasting durations facilitated the estimation of skin hydration levels with an impressive accuracy of around 97%. This application showcases the potential of bio-electrical wearables in non-invasive monitoring for skincare [30]. As another approach to applications of electro-dermal sensors, Almadhor et al.’s proposed federated learning framework for stress prediction showcases the potential for collaborative AI models based on wrist-worn sensor data. Achieving improved stress detection accuracy compared to traditional approaches, this system ensures privacy by training local models before sending parameters to a global model [37].

In the domain of real-time bladder monitoring, Zhang et al. proposed a wearable utilizing bio-impedance data and a random forest machine learning algorithm. Achieving over 90% accuracy in predicting bladder fullness, this system holds promise for aiding in urinary incontinence [12]. To mention another example on the topic, Dheman et al. developed a non-invasive bladder volume estimation system using tetrapolar bio-impedance measurements and a deep learning algorithm. The system uses a wearable sensor node and AI-based artefact suppression to provide quantitative bladder volume measures. The algorithm demonstrated feasibility and comparability to commercial portable ultrasound devices [38].

Finally, in the realm of wireless biomedical monitoring, Yang et al. introduced a non-printed integrated-circuit textile (NIT). This innovative textile, woven with sensors, logic computing, wireless transmission, and power supply, utilizes AI for continuous on-body monitoring and logical codes for emergency assistance. The NIT can monitor body movement, sweat, and light, sending wireless signals for various emergency scenarios. Powered by solar energy harvesting, it serves as a 24/7 private AI nurse [39].

**Figure 4:**
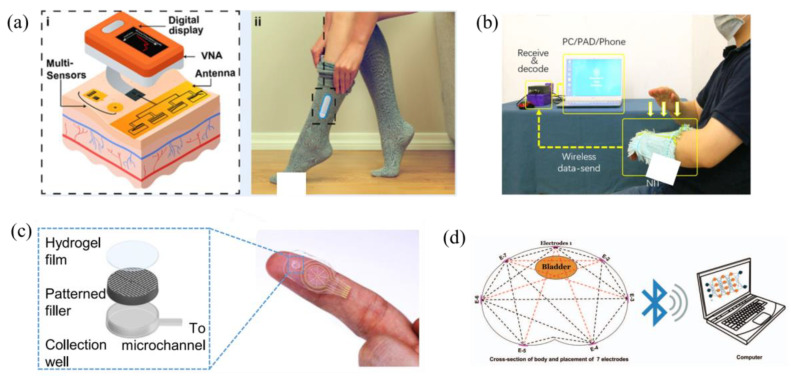
Illustration of examples on bio-impedance and electro-chemical wearables as presented in the literature: (**a**) a wearable system utilizing electromagnetic sensors to non-invasive glucose level measuring [33], (**b**) a non-printed integrated-circuit textile [39], (**c**) a microfluidic patch for continuous sweat analysis [35], and (**d**) an impedance-based wearable for real-time bladder monitoring [12].

Figure 4 provides visual depictions illustrating the practical application of the described wearable technologies. Following this, the subsequent discourse will conduct a comprehensive review of recent advanced research dedicated to implementing bio-impedance and electro-chemical wearable devices through the incorporation of machine learning methodologies.

Among the examined papers on wearable sensors for health monitoring, 75% were identified as employing personalized devices, while the remaining 25% demonstrated potential for personalization (Table 2). The personalized devices utilized a variety of sensors, including photoplethysmography, galvanic skin response, smart textiles, sweat sensors, electromagnetic sensors, printed sensors, and impedance sensors to monitor biomarkers such as blood glucose, respiratory rate, sweat composition, core body temperature, and bladder volume. Reported accuracy ranged from 74.6% to over 99%. The studies spanned publication dates from 2019 to 2023, reflecting the recent progress in the development of personalized wearable health sensors. Overall, this review of the recent literature demonstrates a substantial advancement of personalized wearable devices for continuous health monitoring and their potential to provide individualized care.

## 4. Electro-Mechanical Wearable Devices

The utilization of electro-mechanical elements in wearable devices for the analysis of gait and recognition of motion holds substantial significance in diverse fields, including healthcare, rehabilitation, and robotics. These devices, encompassing soft sensors [40,41] and strain gauges [1,42], facilitate the non-invasive monitoring of human movement patterns, providing invaluable insights for gait analysis and motion tracking. Soft sensors integrated into wearable systems, for example, have the capacity to capture nuanced changes in joint movements and muscle activities, enabling the assessment of gait patterns and the detection of abnormalities in movement. Furthermore, these devices contribute to the creation of intelligent wearable systems, serving purposes such as fall detection, silent communication, and human activity recognition. The incorporation of machine learning algorithms with these wearable devices further amplifies their capabilities, enabling the precise and real-time analysis of gait and motion data. These advancements have the potential to transform personalized healthcare, enhance rehabilitation outcomes, and advance the development of intelligent robotic systems.

Advancing the field of hand gesture recognition, Ferrone et al. developed a wearable wristband equipped with strain sensors. The system utilized strain gauge sensors, machine learning algorithms such as linear discriminant analysis (LDA) and support vector machine (SVM) as well as a leap motion system for validation. Featuring stretchable strain gauge sensors and readout electronics, the wristband achieved a reproducibility of over 98% using the LDA classifier [42]. Another approach was based on an e-textile, where Zeng et al. developed a highly conductive carbon-based e-textile for gesture recognition using heat transfer printing and screen printing. The system uses AI to recognize eight different gestures with 96.58% accuracy [43]. One of the applications of hand gesture recognition was introduced by DelPreto et al., who developed a smart glove with resistive sensors and an accelerometer, using machine learning to classify American Sign Language poses and gestures in real time with high accuracy (96.3%). The system utilizes a strain-sensitive resistive knit for postural information and an accelerometer for motion, with a small custom PCB and microcontroller reading sensors, performing feature extraction, and running a pre-trained neural network [44]. Another interesting application of hand motion detection was introduced during the COVID-19 pandemic. Marullo et al. developed No Face-Touch, a system that uses wearable devices and machine learning to detect hand motions ending in face-touches. The system utilizes a recurrent neural network (RNN) with long short-term memory (LSTM) cells and accelerometer data to detect face-touches, achieving a high true detections rate, low false detection rate, and short time to detect the contact. The system is designed to run on smartwatches and low-cost devices, with a focus on battery consumption and generalization to different users [45]. In silent communication, Smith et al. developed a wearable patch with a graphene-based strain gauge sensor and haptic feedback for silent communication. They used machine learning algorithms, including neural networks, to classify throat movements and predict spoken words with 82% accuracy for movements and 51% for words. They handcrafted a dataset with 15 words and four movements, and used a sensor attached to the throat to collect resistance readings for training and testing the algorithms [1]. In a more recent approach, Tashakori et al. achieved the precise real-time tracking of hand and finger movements using stretchable, washable smart gloves embedded with helical sensor yarns and inertial measurement units. The sensor yarns exhibit a high dynamic range and stability during use and washing. Through multi-stage machine learning, the system achieves low joint-angle estimation errors of 1.21° and 1.45° for intra- and inter-participant validation, matching costly motion-capture cameras’ accuracy. A data augmentation technique enhances robustness to noise, enabling accurate tracking during object interactions and diverse applications, including typing on a simulated keyboard, recognizing dynamic and static gestures from American Sign Language, and object identification [46].

Exploring joint analysis, Gholami et al. developed a fabric-based strain sensor system for knee-joint angle estimation. Implementing machine learning algorithms, including random forest and neural networks, the system processed sensor data and achieved an accuracy of around 6 degrees. The study highlighted the potential applications in healthcare, virtual reality, and robotics [47]. Following knee flexion and adduction moments estimation, Stetter et al. developed an artificial neural network (ANN) using wearable sensors to estimate knee flexion and adduction moments (KFM and KAM) during various locomotion tasks. The ANN was trained with IMU signals and biomechanical data, and the model architecture included two hidden layers with 100 and 20 neurons. The study used a leave-one-subject-out cross-validation method to evaluate the ANN’s performance. The ANN approach does not require musculoskeletal modeling and can provide accurate predictions for new data [48].

Shifting to fall detection, Desai et al. developed a wearable belt using machine learning and signal processing algorithms. With the ability to detect falls within 0.25 s, the system achieved high accuracy using a logistic regression classifier and triggered alerts via a GSM module upon fall detection [49]. In medication adherence monitoring, Cheon et al. utilized sensor data from an Apple Watch to detect low medication states in prescription bottles. Employing machine learning, specifically a gradient-boosted tree model, the system predicted low pill counts with high accuracy and F1 scores. The system involved preprocessing sensor data, extracting summary statistics and training the model using Apache Spark’s MLlib platform [50].

Another interesting application of gait analysis was introduced by Kirsten et al., who developed a sensor-based OCD detection system using AI, personalized federated learning, and motion sensors. The system achieved high AUPRCs and demonstrated privacy-preserving model training [14]. Meanwhile, Chee et al. explored gait analysis and machine learning for diabetes detection. They emphasized the potential of deep learning models like CNN and LSTM in analyzing gait data. The paper highlights the use of gait sensors and features, as well as the need to implement DL models for improved accuracy [11]. In another approach, Igene et al.’s SVM model, utilizing accelerometer data, showcased an accuracy of 94.4% in predicting Parkinson’s disease. Employing ANOVA, PCA, and grid search for feature selection and hyperparameter tuning, this application emphasizes the potential of electro-mechanical wearables in early disease detection and monitoring [29]. Li et al. developed a multimodal sensor glove to assess Parkinson’s disease symptoms in patients’ hands. They used various algorithms to process signals, achieving a 95.83% accuracy in identifying tremor signals. The glove assessed flexibility, muscle strength, and stability, showing high consistency with clinical observations. The system’s reliability was confirmed through repeated experiments, with intraclass correlation coefficients exceeding 0.9 [51].

Moving to stretchable sensors, Nguyen et al. developed a stretchable gold nanowire sensor for motion tracking. They used a machine learning algorithm to characterize the sensor’s response, achieving a high gauge factor of 12 and an error of less than 2 degrees in measuring bending motion [52]. As another example on stretchable sensors, Feng et al. developed a sensing-actuation unit for force estimation in soft stretch sensors. They used deep learning methods, including LSTM and Informer, to calibrate and predict force, achieving a mean square error (MSE) of less than 0.28 N^2^ and normalized root mean square error (NRMSE) of less than 2.0%. The unit has adjustable stiffness and is promising for applications like lightweight flexible exoskeletons [53]. Another soft sensor for gait generation was introduced by Kim et al., who introduced a semi-supervised deep learning model using microfluidic soft sensors. Leveraging a deep autoencoder, the model embedded gait motion into a latent motion manifold, reducing the need for a large calibration dataset. The system utilized AI to generate natural human gait motion from sensor outputs [40]. Transitioning to upper-limb posture detection, Giorgino et al. introduced a system utilizing conductive elastomer sensors for neurological rehabilitation. Employing machine learning for posture classification, the system addressed challenges related to sensor noise and generalization, achieving high recognition performance for real-time classification [54].

Exploring material surface recognition, Liu et al. developed smart gloves with ZNS-01 sensors to recognize five material surfaces. They used machine learning algorithms like XGBoost to achieve 98% classification accuracy. The system extracts time and frequency domain characteristics to train the models [55]. Introducing an advanced system for the ongoing wireless monitoring of arterial blood pressure, this technology, created by Li et al., features a thin, soft, and miniaturized design. The system incorporates a sensing module, active pressure adaptation module, and data processing module to identify the blood pulse wave, apply back pressure, and extract the pulse transit time interval. Employing a sophisticated multiple-feature fusion framework and ensemble learning, particularly extreme gradient boosting, the system constructs an estimation model. This model integration includes AI techniques, ensuring meticulous control over blood pressure [56].

Shifting to body movement detection, Wang et al. developed a wearable plastic optical fiber sensing system for human motion recognition using machine learning. The system uses AI, such as support vector machines and convolutional neural networks, to analyze motion signals and achieve high recognition accuracy. The system’s key parameters include feature vectors, cumulative contribution rate, and time consumed for recognition [2]. On another approach, Mani et al. developed a conductive fabric-based suspender system for human activity recognition (HAR) using machine learning and deep learning techniques. The system achieved an accuracy of 98.11% using eight different classifiers, including KNN, SVM, RF, and LSTM [57]. Utilizing MXene technology, Yang et al. developed wearable Ti_3_C_2_T_x_ MXene sensor modules with in-sensor machine learning (ML) models for full-body motion classifications and avatar reconstruction. The sensors exhibited ultrahigh sensitivities within user-designated working windows, and the ML chip enabled in-sensor reconstruction of high-precision avatar animations with an average error of 3.5 cm. The ML models achieved 100% accuracy for full-body motion classification without using image/video data. The edge sensor module with ML chip allowed the real-time and high-accuracy determination of 15 avatar joint locations, leading to personalized avatar animations. The integration of wearable sensors with ML chip for in-sensor machine learning and avatar reconstruction is a significant advancement in the field of wearable sensors and human–machine interaction [58]. Jiang et al. summarized the benefits of using advanced algorithms in wearable tactile sensors, including time series models and classification algorithms based on machine learning and signal processing. They discussed the integration of AI in the system, including the use of machine learning for motion recognition and voice recognition [59].

Vasdekis et al. developed WeMoD, an AI-based approach for predicting daily step count and setting personalized physical activity goals using a combination of physiological, psychological, and contextual features. They utilized ML algorithms such as ridge regression, decision tree, random forest, and gradient boosting regressor to achieve a mean absolute error of 1908 steps [8]. Papaleonidas et al.’s focus on high-accuracy human activity recognition models introduces the potential for health monitoring and smart home management. Utilizing machine learning and raw signals from wearables, the model achieved 99.9% accuracy. The integration of ML algorithms and variable segmentation methodology showcases the versatility of electro-mechanical wearables in recognizing activities [60].

**Figure 5:**
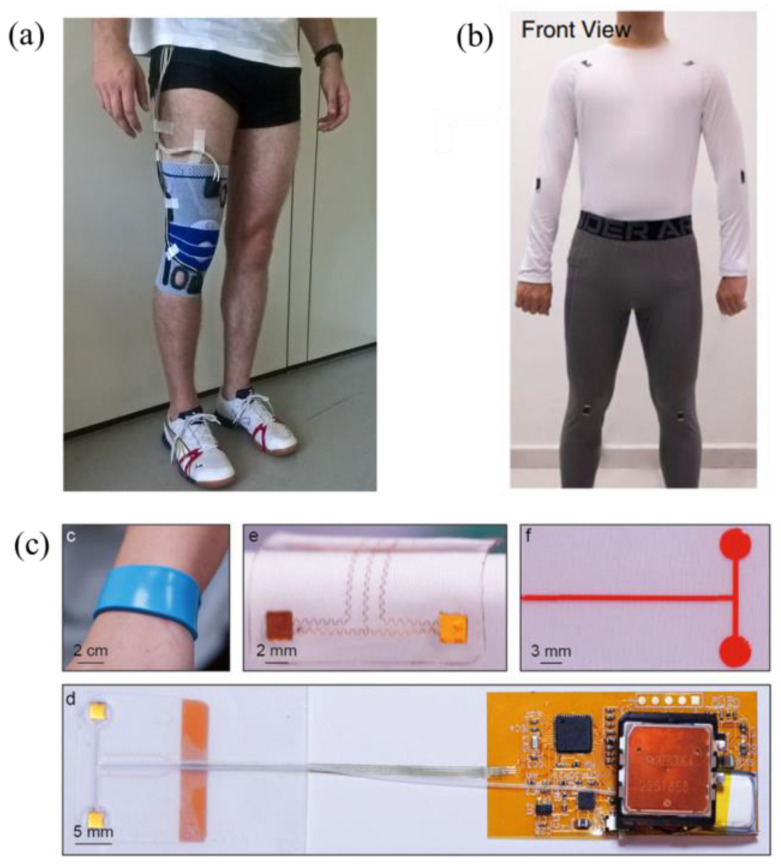
Setup for various electro-mechanical wearable devices: (**a**) a set of wearable sensors to estimate knee flexion [48], (**b**) a thin, soft, and miniaturized design for arterial blood pressure [56], and (**c**) Ti_3_C_2_T_x_ MXene sensor modules for full-body motion classifications [58].

Visual representations exemplifying the application of these wearable technologies are presented in Figure 5. The subsequent discussion will delve into a review of recent advanced research focused on the implementation of electro-mechanical wearable devices utilizing machine learning techniques.

Table 3 reviews recent progress in wearable sensors for personalized health monitoring. Among the 23 papers reviewed, 78% (18 papers) demonstrated wearable devices personalized for individual users, while 22% (5 papers) showed potential for personalization but did not implement it. The personalized devices targeted a wide range of applications including posture, gesture, and motion tracking; fall detection; medication monitoring; knee and joint movement; touch sensing; muscle activity; step counting; blood pressure; and diabetes detection. Sensing modalities included strain gauges, stretchable sensors, microfluidics, IMUs, EMG, tactile sensors, and optical fibers. Reported accuracy ranged from 75–100%, with 78% of papers achieving over 90% accuracy. The high accuracy and focus on personalization in the majority of surveyed devices highlights the growing ability of wearable sensors to provide customized real-time health insights for individual users. This progress suggests personalized wearable health monitoring will continue expanding in the coming years.

## 5. Conclusions

In conclusion, recent strides in wearable device development underscore the integration of machine learning algorithms, ushering in a new era of personalized health monitoring and intervention systems. The comprehensive review of literature in this field, with a particular emphasis on personalized wearables, revealed a noteworthy finding: 78.5% of the scrutinized articles showcased the incorporation of personalized features, while the remaining articles demonstrated the potential for personalization. These wearable systems leverage sophisticated algorithms to process diverse sensor data, ranging from strain gauges to electrochemical sensors, enabling the provision of tailored health insights and interventions. The application of machine learning extends beyond general health monitoring, delving into personalized healthcare solutions for specific medical conditions such as diabetes, sleep disorders, and neurological rehabilitation. The analytical power of AI facilitates the interpretation of physiological signals, the prediction of disease states, and the delivery of personalized interventions, thereby enhancing disease management and patient outcomes. Furthermore, the development of personalized wearables addresses specific health challenges, exemplified by conditions like urinary incontinence, panic attacks, and obsessive compulsive disorder. Machine learning algorithms play a pivotal role in detecting and predicting these conditions, enabling early intervention and personalized support. These collective advancements underscore the immense potential of personalized wearable devices in catering to individual health needs and ultimately elevating the quality of life for individuals with diverse health conditions.

## Figures and Tables

**Table 1:** Summary of the reviewed papers with bio-electrical sensors.

Ref.	Medical Application	Type of Sensor	Type of Input Data	Use of ML	Type of ML Model	ACC. *	PERSON. *	Year
[10]	Personalized deep brain stimulation for Parkinson’s patients	BioStamp nPoint	Inertial sensor data	Classify deep brain stimulation parameters	MLP	95%	Yes	2020
[25]	Non-invasive seizure forecasting	E4, Empatica	EDA, accelerometer, BVP, temperature	Classify seizure periods from non-seizure periods	LSTM	N/A	Yes	2020
[19]	Personalized lifestyle recommendations to improve blood pressure	Fitbit Charge HR and Omron Evolv	HR, sleep activity, number of steps	Classify input data and identify the most important lifestyle factors that impact BP trend	RF	N/A	Yes	2021
[21]	Prediction of blood glucose level	Zephyr BioHarness3	ECG, glucose level	Regression of the input data was carried out using LGBM, GBR, AdaBoost, and linear and ridge regressors	LGBM, GBR	N/A	Yes	2022
[13]	Seven-day panic attack prediction	Garmin Viívosmart 4	Sleep, HR, activity level, AQI	Classify and predict panic attack	RF, LDA	67.4~81.3%.	Yes	2022
[7]	Monitoring stress level	E4, Empatica	EDA, ECG, PPG	To classify stress and non-stress status prediction	SVM. KNN, RF, NB, LR	86.4%	Yes	2022
[18]	Monitoring the rehabilitation phase and its effectiveness on hemiplegic ankle patients	Smartphone gyroscope	Max, min, mean, standard deviation, and CV of gyro signal	Classify gyro data and distinguish between the initial phase and the final phase of therapy regimen	SVM	91.7%	Yes	2022
[22]	Prediction and prevention of heat stroke in hot environments	pHST meter, HR monitor, accelerometer	pHST, heart rate, acceleration data	Classify the pHST parameter to predict heat stroke	KNN	85.2%	Yes	2022
[6]	Estimating BP and categorizing it to (normal, pretension, and hypertension)	ECG sensor	ECG	Classification of ECG data with XGBoost to estimate BP and categorize. Regression by ANN	ANN, XGBoost	73.37%.	No	2022
[24]	Detection of pill intake for patients with dementia-related conditions	LILYGO^®^ TTGO T-Watch	Acceleration data	Supervised learning to detect three types of hand gestures: pill intake, casual hand movement, and still hand	three-layer NN	99%	Yes	2022
[20]	Real-time monitoring and interpreting RR interval data	Polar H10	ECG	Classify data into five categories: normal, supraventricular, ventricular ectopic, fusion, and unknown	SVM, decision tree	96%	Yes	2022
[16]	Real-time emotion recognition	Behind-the-ear EEG sensor	EEG	Classify EEG data into two emotional states: positive states and negative states	SVM, MLP, 1D-CNN	94.87%	Yes	2023
[9]	Detection of sleep apnea	Single-lead ECG sensor	ECG	Classify ECG data to recognize sleep apnea	Voting classifier	88.13%	No	2023
[26]	Personalized detection of seizure using HRV	wearable ECG device	ECG, HR variability, EEG	Patient-adaptive LRML was used to classify HRV data according to the video-EEG to detect seizure	LRML	78.2%	Yes	2023
[23]	Measuring stress level	Empatica E4	PPG, EDA	Binary classification of PPG and EDA using RF, SVM, and LR algorithms	RF, SVM, LR	90%	Yes	2023

* ACC.: accuracy of the proposed wearable system. PERSON.: whether the proposed system is defined as a personalized wearable system or not.

**Table 2:** Synopsis of wearable devices employing electro-chemical and bio-impedance technologies.

Ref.	Medical Application	Type of Sensor	Type of Input Data	Use of ML	Type of ML Model	ACC.	PERSON.	Year
[5]	Estimation of blood glucose level	PPG and GSR sensor	PPG, GSR	Used deep neural network for feature extraction and regression.	1D-CNN	80%	Yes	2019
[31]	Analyzing cell based-on impedance flowcytometry	Microfluidic impedance meter	Basic impedances in microfluidic channel	NARX model was used to extract features from the channel impedance as combination of basis impedances.	NARX	99.99%	No	2020
[32]	Respiratory monitoring	Biopac belt, ECG sensor	Thoracic bio-impedance, ECG	Classifying BioZ into clean and noisy classes, carried out by SVM. Then, TL was used to optimize each of the classifiers and to obtain an adapted model of CNN for each breathing type.	TL, SVM, CNN	N/A	No	2021
[39]	As an AI nursery assistant to monitor body health and environment	Smart textile with sweat, motion, and light intensity sensor	Sweat, motion, and light intensity	N/A	N/A	N/A	Yes	2021
[3]	Measurement of sweat glucose level	Electrochemical sweat sensor	Impedance, relative humidity, temperature	Estimation of glucose level based on raw impedance, humidity level and temperature.	SL, decision tree	94%	Yes	2022
[30]	Monitoring hydration level on the skin	GSR sensor	GSR	Classifying the GSR data to three hydration states, namely hydrated, mildly dehydrated, extremely dehydrated, along with three posture types, namely sitting, standing, and walking.	Hybrid Bi-LSTM	97.83%	Yes	2022
[33]	Personalized and non-invasive monitoring of blood glucose level	Body-matched electromagnetic sensor	EM scattering parameters, ambient and skin temperature, humidity level	Gaussian parametric regression (GPR) was used to estimate blood glucose level based on the selected parameters.	GPR	99.01%	Yes	2022
[28]	Monitoring wound healing	MXENE-attached wound bandage (SMART-WD)	pH	Recognition of the healing stage of the wound.	Deep ANN	94.6%	Yes	2022
[37]	Monitoring and detecting stress	EDA sensor	EDA	Classify the input data into five categories: transient, baseline, stress, amusement, and meditation.	DNN	86.82%	Yes	2023
[34]	Monitoring core-body temperature	Printed electrochemical sensors embedded into a plastic microfluidic sweat collector	Na^+^ and K^+^ printed sensor	Linear regression (LR), support vector regression (SVR), and random forest regression (RFR) were used to estimate core body temperature.	LR, SVR, RFR	>99%	Yes	2023
[12]	Monitor and analyze bladder monitor	Bio-impedance meter	Impedance of the bladder region	Determination of the urination status using RF algorithm.	RF, SVM, DNN	>90%	Yes	2023
[38]	Bladder level	Bio-impedance meter	Lower abdomen impedance	Using SVM and DNN to estimate the bladder volume quantitatively and remove artefacts.	SVM, DNN	74.6~84.8%	No	2023

**Table 3:** Overview of wearable devices employing electro-mechanical technology.

Ref.	Application	Type of Sensor	Type of Input Data	Use of ML	Type of ML Model	ACC.	PERSON.	Year
[54]	Posture recognition and rehabilitation exercise monitoring	Strain sensor attached to the upper body	Signals from strain sensors	Logistic regression was used to recover the current body posture from the sensor reading	LR	75%	Yes	2006
[42]	Monitoring and rehabilitating hand gestures	Stretchable strain gauge	Signals from strain sensors	LDA and SVM were used for evaluating the performance of the system with the collected data	LDA, SVM	98%	Yes	2016
[47]	In-home rehabilitation and long-term tracking of movements of people with knee disorders	Fabric-based strain sensor	Signals from strain sensors sync with camera	NN and RF were used for estimating the knee joint angle based on the strain sensor data	NN, RF	97%	No	2018
[61]	Movement and gesture detection	Piezoresistive woven wool glove	Signal from piezoresistive sensor	Data pre-processing and gesture recognition was carried out by SVM	SVM	97.8%	No	2019
[40]	Assistive human walking in rehabilitation	Microfluidic-based stretchable sensor	Sensor output, position vector	Semi-supervised deep learning model including a deep auto-encoder and components such as sequential encoder networks, alignment networks, and motion representation networks	Semi-supervised DNN	N/A	Yes	2019
[49]	Detection of falls in elderly people, triggering an alert, taking immediate action (e.g., airbag)	Gyroscope, accelerometer	Gyro and acceleration data	Utilizing logistic regression, falling incidents were identified, taking into account all overlooked data during sensor thresholding.	LR	100%	Yes	2020
[50]	Detect low medication state in the container and notify a medical system, doctor, or pharmacy.	Apple Watch	Gyroscope, accelerometer, audio decibel levels, and labels indicating the number of pills	Specifically comprising 200 estimators using a tree-depth of three used for detecting low counts of pill medication in standard prescription bottles.	Gradient Boosted Tree machine	80.27%	No	2020
[48]	Provide valuable biofeedback systems for knee osteoarthritis (KOA) patients	IMUs located on the right thigh and shank	IMU signals	ANN was used to estimate KFM and KAM during various locomotion tasks	ANN	N/A	Yes	2020
[59]	Health status monitoring, social interactions evaluation, disability assistance, baby crying, respiratory monitoring for infants, etc.	Tactile sensor	Signals from tactile sensors	To classify the signal and output the judgment results (effective in complex movements)	SVM, DNN	N/A	Yes	2021
[14]	Early detection of obsessive compulsive disorder (OCD)	IMU attached to the left and right arm	Gyro and acceleration data	For evaluating personalized federated learning algorithms and non-collaborative training algorithms	LSTM	90%	Yes	2021
[45]	Hand motion	IMU of a smartwatch	acceleration data with synced video	Used for classification tasks with unbalanced datasets	RNN, LSTM	N/A	Yes	2021
[55]	Smart glove with the ability of distinguishing different materials	Ultra-thin, and stretchable ZNS-01 sensor	Touch force data	Used for recognizing five different material surfaces	XGBoost	98%	Yes	2021
[52]	Evaluation and improvement of human skills proficiency such as medical skills	Stretchable gold nano-wire	Output data of the sensor	Used to enhance the predictability of the sensing response of the developed sensor	LSTM	~99%	Yes	2021
[1]	Silent communication for individuals with speech and hearing impairments	Graphene strain gauge sensor	Sensor output data	Used for automated classification of input signals	NN, LSTM	82%	Yes	2021
[60]	Monitoring the movements of elderly individuals in hospital rooms	RespiBAN and Empatica E4	IMU data, BVP, EDA data, temperature	Gaussian support vector machine was used for human activity recognition	KNN, GSVM	99.9%	Yes	2022
[8]	Boosting physical activity levels through personalized self-monitoring and coaching	Gyroscope and IMU	Daily physical activity	Supervised ML regression algorithms was used to predict daily step count and set goal	DT, RF, GBR	N/A	Yes	2022
[58]	Full-body avatar reconstruction	MXene-based strain sensor	Signals from strain sensors	In-sensor machine learning model, specifically implemented on an ML chip, for the determination of full-body avatar joint locations		100%	Yes	2022
[44]	Improve communication for individuals who use American Sign Language (ASL)	Smart glove, accelerometer	Strain and acceleration data	Classifying sign language poses and gestures in real time	LSTM	96.3%	No	2022
[56]	Continuous wireless monitoring of ambulatory artery blood pressure for preventing and diagnosing hypertension-related diseases	Conformal piezoelectric sensor array	Output of the piezoelectric sensors	Classify the input data to detect the blood pulse wave, pulse transit time interval, and other physiological features and local pulse wave velocity (PWV).	XGBoost	98% (err < 5 mmHg)	Yes	2023
[2]	Health monitoring, motion analysis, activity monitoring of the elderly, and identifying falls	Optical fiber-based wearable motion detection system	Data from optical receiver module	For classification and recognition of motion, based on the data from optical system	SVM, MobileNetV2 network, transfer learning	>98.28%	Yes	2023
[29]	Early prediction of Parkinson’s disease	Accelerometer	Accelerometer sensor signals	SVM classifier was used for the automatic detection of Parkinson’s disease based on daily movement data.	SVM	94.4%	Yes	2023
[43]	Monitoring of joint motion and recognition of different gestures	Highly conductive carbon-based e-textile	Signal data from the wearable device	ANN was used for the classification and recognition of different gestures	ANN	96.58%	Yes	2023
[53]	Active rehabilitation, walking assistance, and continuous human movement monitoring	Capacitive soft stretchable sensor	Electrical and mechanical properties of the sensor	LSTM and Informer were used for force calibration and prediction in the paper.	LSTM, Informer	>98%	No	2023
[11]	Detection of diabetes using human gait analysis	Kinematic and kinetic sensors such as accelerometers, shoe-type IMUs, ear-worn inertial sensors, motion capture systems, force-measuring shoes, pressure sensors, EMG sensors	IMU and accelerometer data, EMG, motion capture systems data, signals from force-measuring shoes and pressure sensors	A combination of various machine learning models including SVM, KNN, RF, DNN, CNN, MLP, and LSTM was used to detect diabetes	SVM, KNN, RF, DNN, CNN, MLP, LSTM	98.68%	Yes	2023
[57]	Human activity monitoring and identification	Conductive fabric-based suspender	Data from sensor output	A variety of classifiers were applied to extract human activity from the sensor data	KNN, SVM, LSTM, RF, LR, DT, GBDT	98.11%	Yes	2023
